# Effect of Coal-Derived Graphene Oxide on the Mechanical and Microstructural Characteristics of Concrete

**DOI:** 10.3390/ma18122774

**Published:** 2025-06-12

**Authors:** Iftekhar Dipta, Kam Ng, Jacob Chadwick, Chooi Kim Lau, Hua Yu, Patrick Alfred Johnson

**Affiliations:** 1 Department of Civil and Architectural Engineering and Construction Management, University of Wyoming, 1000 E. University Avenue, Laramie, WY 82071, USA; idipta@uwyo.edu (I.D.); clau1@uwyo.edu (C.K.L.); 2 Department of Chemical Engineering, University of Wyoming, 1000 E. University Avenue, Laramie, WY 82071, USA; jchadwi2@uwyo.edu; 3 Department of Civil and Construction Engineering and Management, University of Texas at Tyler, 3900 University Blvd, Tyler, TX 75799, USA; hyu@uttyler.edu; 4 Department of Materials Science and Engineering, Iowa State University, Ames, IA 50011, USA; paj3@iastate.edu

**Keywords:** coal-derived graphene oxide, concrete, mechanical properties, microstructure, cement

## Abstract

Past studies have offered insights into how graphite-derived graphene oxide (GDGO) can improve the mechanical properties and alter microstructural characteristics of concrete. These advantages can significantly impact the construction industry regarding cost, sustainability, and efficiency. However, the high cost of GDGO can make commercial implementation unattainable. This paper comprehensively investigates coal-derived GO as a cost-saving alternative to commercial GDGO while achieving comparable concrete performance. Different GO proportions were incorporated into concrete mixes through laboratory experiments to determine the effect on mechanical properties and microstructures. In this research, concrete mixes were formulated by replacing a portion of cement with coal-derived GO and adding this GO as an additive to concrete at varying percentages (0.05%, 0.10%, 0.25%, 0.50%, 1%, and 1.5% by weight of cement). The study revealed flexural, split tensile, and compressive strength improvements of 3.3%, 2.3%, and 21.2%, respectively, at a minimal 0.05 wt.% GO replacement. Optimal inclusions of GO as an additive ranging from 0.05 to 0.25 wt.% were identified to exhibit a maximum increase in mechanical properties. More precisely, adding 0.10 weight percent of GO as an additive to concrete showed increases in flexural, split tensile, and compressive strengths of 14.05%, 9.7%, and 34.2%, respectively. Furthermore, detailed analyses, including modulus of elasticity, Poisson’s ratio, heat of hydration, and microstructural analysis provided comprehensive insights into the enhanced mechanical performance of GO-incorporated concrete. Additionally, the study revealed a lower Ca/Si ratio in GO concrete, further validating the reinforcing properties of the GO.

## 1. Introduction

Cement, the fundamental binding material in concrete [1], has traditionally suffered from brittleness and low tensile strength [2]. Numerous endeavors have been undertaken to improve its properties and performance by incorporating supplementary cementitious materials (e.g., fly ash and blast-furnace slag) [3], and reinforcement (e.g., glass and steel) [4]. Moreover, it has become increasingly evident in recent years that the construction industry is among the sectors with significant concerns regarding carbon dioxide (CO_2_) emissions. According to estimates, producing one ton of ordinary Portland cement releases approximately one ton of CO_2_ into the atmosphere [5]. Therefore, pursuing the feasibility of substituting even a small amount of cement with alternatives to promote environmentally friendly practices is of great interest.

Graphene oxide (GO), a two-dimensional carbon-based material [6] that exhibits a large and well-defined surface area, coupled with outstanding mechanical strength [7] and impressive thermal conductivity [8], has garnered significant attention in recent years for its potential to enhance the properties of cement composites [9], especially concrete. As researchers strive to develop sustainable and high-performance construction materials [10], incorporating GO as an additive to concrete and partial replacement for cement holds promise in revolutionizing the construction industry. Similar to graphite, GO possesses an aromatic structure characteristic of other graphene-based derivatives. However, as a result of chemical reactions, certain covalent bonds are cleaved, leading to the attachment of functional groups, such as epoxy, carbonyl, hydroxyl, and phenol [11] onto the one-atom-thick *sp^2^* aromatic monolayer structure [12]. The inclusion of these functionalities in concrete offers distinct advantages. Due to its substantial specific surface area and high aspect ratios, GO is distinguished among nanomaterials for its exceptional hydrophilicity [13]. Its oxygen-containing functional groups allow it to disperse readily in the concrete mix [14]. The hydration characteristics of concrete-GO mixtures produce calcium silicate hydrate (CSH) gel in micropores. The mechanism behind the strengthening effect on concrete has been unveiled by filling up the micropores with CSH, demonstrating the positive impact of GO on the performance of concrete. The introduction of GO to 0.4% by wt. of cement as a replacement leads to improved hydration by enhancing the ion mobility into the cement paste, which increases the interaction between Ca^2+^ and cement particle surfaces, facilitating the nucleation and development of CSH on cement surfaces. This ultimately yields a more resilient and durable final product [15]. Adding 0.04% GO to the concrete increased compressive strength up to 15.1% [16]. Similarly, the introduction of 0.05% GO by weight of cement was reported to significantly improve the compressive strength by 25–33% and enhance the flexural strength by 41–59% of GO-concrete [7]. Another study observed that incorporating 0.06% GO by weight of cement into concrete led to a remarkable increase in compressive strength by 72.7%. Including 0.04% GO significantly increased flexural strength by 67.1% compared to regular concrete without GO [17]. Oxygenated functionalities on the GO surface facilitate closer interaction with the cement particles, effectively serving as nucleation sites for the cement phases. As a result, this encourages and enhances the reaction between cement and water, leading to a more rapid and efficient hydration process [18]. The observation suggests that the leaching of calcium hydroxide (CH) during the hydration stages is notably enhanced, particularly evident at the 28-day curing age [19]. Table 1 summarizes various studies on adding GO to concrete, indicating the percentage of GO by weight, water-cement ratio, and the corresponding compressive strength results. These findings highlight the diverse effects of GO on concrete properties across different studies.

Thus, GO emerged as the most promising candidate for this research endeavor. However, using GO in concrete possesses some challenges. The primary challenge identified by various sources includes the exorbitant cost of commercial GO ranges between $75–200/g [27], which restricts most research efforts to a narrow GO range of 0.03% to 0.1% [28]. In the past, there has been a lack of emphasis on developing cost-effective methods for producing GO. Additionally, the current techniques for GO production are primarily geared towards academic or small-scale projects, typically involving quantities ranging from grams to tens of grams. While these quantities suffice for educational purposes, they may fail to meet commercial demands.

While past research has explored the use of GO produced from different techniques for synthesizing GO from nature and waste graphite [29], limited attention has been given to applying coal-derived GO in concrete. In previous work, we demonstrated cost-effective synthesis of GO from coal with properties comparable to commercially sourced GO derived from graphite [30]. Prior studies demonstrated that the direct use of coal char was incorporated into concrete at 0.5% by weight of cement, resulting in a 28% increase in compressive strength [31]. This finding serves as the foundation for utilizing this coal as a raw material for GO in concrete. Hence, this research addresses this knowledge gap by investigating the effects of different coal-derived GO contents on the mechanical properties and microstructures of concrete. To overcome the challenges as mentioned earlier, this research explores the impact of incorporating low-cost coal-based GO as an additive and cement replacer into concrete at varying percentages (0.05, 0.1, 0.25, 0.5, 0.75, 1.0, 1.5) by weight of cement. Considering different GO contents, this study assesses its impact on workability, compressive strength, flexural strength, tensile strength, modulus of elasticity, Poisson’s ratio, the heat of hydration, microstructural characteristics, and overall quality of the coal-derived GO-modified concrete. This study lies in its potential to uncover novel insights into the performance of low-cost coal-derived GO-modified concrete, ultimately contributing to developing sustainable and high-strength construction materials with diverse applications in the construction industry.

## 2. Raw Materials

### 2.1. GO

**Figure 1:**
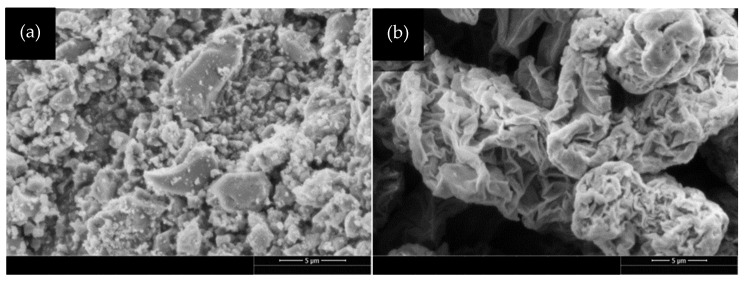
SEM image of: (**a**) coal-derived GO, (**b**) commercial GO. Scale: 5 µm.

GO possesses both *sp^2^* and *sp^3^* hybridization [32] and is a precursor in various graphene manufacturing techniques. It shares a resemblance to graphene, displaying the characteristic honeycomb structure. However, one notable distinction lies in the presence of numerous oxygen functional groups along the edges of its honeycomb structure. These functional groups, including hydroxyl (–OH), carboxyl (–COOH), epoxy (C–O–C), and carbonyl (C=O), are commonly found in GO. In this study, GO is chemically processed from Powder River Basin (PRB) coal [33], Wyoming, which is classified as sub-bituminous coal. With 12 active mines, this PRB is the largest coal region in Wyoming, USA [34]. The synthesis of GO begins with pyrolyzing the PRB coal in a furnace at temperatures up to 850 °C to remove volatiles and tar, leaving behind coal char. This coal char is treated with nitric acid to oxidize aromatic carbons and reduce ash content, forming low-cost GO [30]. The GO was thoroughly washed until the filtrate reached a pH of ~6.5. The final GO, when dispersed in water, exhibited a pH of ~3.0 due to its surface functional groups. In addition to the nitric acid method, GO can be produced from coal using various other techniques, including Hummer’s method [35], improved Hummer’s method [36], and modified Hummer’s method [37]. The University of Wyoming has been awarded a patent for the nitric acid method of producing GO from coal (U.S. Patent No. UW 2021/0214231 A1, 2021) [38]. The SEM image of lab-produced coal-derived GO is shown in Figure 1a. Considering the difficulty in accurately capturing the total powder sample by individual microscopy sampling, Figure 1a indicates that GO produced from coal appears composed of discrete particles covering a wide range of sizes. Most particles, with very few exceptions, are less than five μm, giving the composition a modest irregularity but a noticeable increase in the number of larger particles clumped together. This microstructure of coal-derived GO is different from commercially available GO. Commercial GO (ACS Material, Pasadena, CA, USA) depicts a structure resembling crumpled paper with spaces between the particles, the majority of which are larger than five μm, shown in Figure 1b. 

Table 2 shows the physical and chemical properties of coal-derived and commercial GO. The dispersion characteristics of GO in water can vary significantly due to their inherent differences in size and shape. Coal-derived GO, characterized by a smaller lateral size of 0.3 to 1 μm, forms micron-sized powders. These smaller particles, with a higher surface area, interact more effectively with water molecules and may promote better dispersion. In contrast, commercial GO, with a lateral size ranging from 0.2 to 10 μm (see Table 2), typically exists as nanosheets. These larger, flat structures tend to stack or aggregate, making even dispersion in water more challenging [39]. The coal-derived GO is washed to a pH of about 3.0, so it technically should be more acidic. Furthermore, coal-derived GO showcases a significantly higher carbon content (61.91%) and lower oxygen content (33.68%) than commercial GO (see Table 2). The carbon-to-oxygen ratio is higher in coal-derived GO at 1.84, suggesting a distinct composition. This higher carbon content and lower oxygen content retain a more robust *sp^2^*-bonded carbon network characteristic of graphene, contributing to greater mechanical reinforcement and improved strength and durability of the concrete. Moreover, coal-derived GO is characterized by a higher ash content (8.73%) and moisture content (8.03%), influencing its workability of concrete. These distinctions create differences between the two GOs and provide this study with information about the usage of coal-derived GO in concrete.

### 2.2. Cement

Quikrete ordinary Portland cement (Type I/II) (The QUIKRETE Companies, Atlanta, GA, USA) is used in this study in all concrete experiments [40]. This ordinary Portland cement (OPC) has a specific gravity of 3.15 relative to water. The chemical composition of cement is given in Table 3, that reflects the oxide content in this cement, with key constituents such as silicon dioxide (SiO_2_), calcium oxide (CaO), and sulfur trioxide (SO_3_) contributing to its quality and properties.

**Figure 2:**
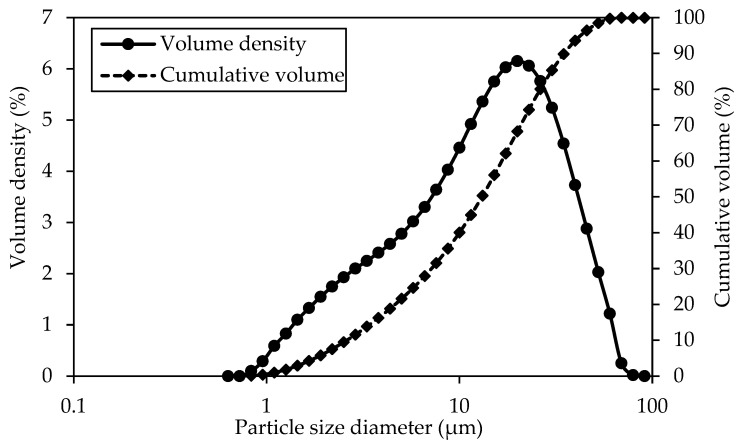
Quikrete OPC (Type I/II) particle size distribution.

The cement above exhibits a specific surface area of 0.903 m^2^/g. Figure 2 illustrates the particle size distribution for this particular cement, showcasing an average particle size ranging from 1 to 100 μm, which is higher than coal-derived GO (0.3–1 μm).

### 2.3. Aggregates

#### 2.3.1. Coarse Aggregate (CA)

Concrete’s strength, durability, and volume are all influenced by the coarse aggregate (CA). As CA contributes the highest volume in the concrete mix, it impacts the mix design. According to ACI mix design (ACI 211.1-91) [41], the amount of water necessary to get workability and the amount of fine aggregate needed to create a unified mix are determined by the maximum size of the CA. Concrete’s workability, durability, and cement and water requirements are all impacted by the grading, particle-size distribution, specific gravity, unit weight, absorption capacity, and moisture content of the CA. Another factor to consider is the shape of the CA; angular shapes are recommended for high-strength concrete, while rounder shapes are more cost-effective for regular-strength concrete. This study uses crushed angular-shaped stones from the local supplier in Laramie, WY, USA, as CA. The CAs are sieved with a maximum size of 9.5 mm for preparing 50 mm diameter by 100 mm height cylindrical concrete samples.

#### 2.3.2. Fine Aggregate (FA)

This study uses local sand, supplied by the same local source as the coarse aggregate in Laramie, WY, USA, as the fine aggregate (FA). The standard sieve analysis revealed that the fineness modulus (FM) is 2.58. The unit weight of 1586.68 kg/m^3^ is determined using the rodding method. The aggregate size, the bulk specific gravity of both oven-dry (OD) and saturated surface dry (SSD) conditions, apparent specific gravity, dry rodded unit weight, absorption capacity, moisture content, and FM are summarized in Table 4.

## 3. Design Mix of Concrete Samples

### 3.1. Concrete Specimens

Cylindrical concrete specimens with 50 mm diameter by 100 mm length were prepared for compressive tests according to ASTM C39 [47] and indirect split tensile strength tests by ASTM C496 [48]. Concrete beam samples measuring 100 mm by 100 mm by 355 mm were prepared for flexure strength according to ASTM C78 [49]. These specimens were cured for 3, 7, 14, 28, and 56 days for compressive strength tests and 28 days for split tensile and flexural strength tests. All specimens were subjected to sealed curing conditions until the designated testing age.

### 3.2. Concrete Design Mix Proportions

Past research studies mainly investigated the effect of adding GO as a replacement to cement on concrete performance [28]. This study focuses on a broader range of GO contents and compares the effect of both GO as a cement replacement and GO as a concrete additive on improving performance.

#### 3.2.1. GO as a Cement Replacement

GO is added as the partial cement replacement in the concrete mix design with a target compressive strength of 30 MPa as the first designation without GO and the second designation based on 50-mm diameter by 100-mm high specimens (i.e., C30-GO0-50). Eight concrete mixes for GO as a replacement, summarized in Table 5, are prepared to investigate the engineering properties of concrete. The different cement replacements by GO are 0%, 0.05%, 0.10%, 0.25%, 0.50%, 0.75%, 1%, and 1.5%, which are denoted as GO0, GO5, GO10, GO25, GO50, GO75, GO100, and GO150. Past studies considered water-cement (w/c) ratios between 0.20 and 0.66 [28]. As GO has a high water absorption capacity [50], and in concrete, water is the main ingredient to react with cement, we chose a high water-cement ratio for this study. Moreover, very few studies have been done on the effect of a high water-cement ratio in GO-concrete [51]. According to Peng et al. (2019) [52], a higher water-to-cement ratio facilitates the dispersion of GO, enhancing its positive effect on the strength and microstructure of hardened cement paste. This study maintains the concrete mixes at a constant water-to-binder (w/b) ratio of 0.60 with a selected mix proportion of 1:2.3:2.0 [(Cement + GO):FA:CA] by weight. Here, the binder refers to the total mass of cement and GO combined.

#### 3.2.2. GO as a Concrete Additive

GO is added as an additive to concrete mixes, as summarized in Table 6. For this case, the water-to-cement (w/c) ratio was maintained at 0.60 across all mixtures, while the total binder content increased effectively with the increasing GO dosage. The mix proportion remained at 1:2.3:2.0 (Cement:FA:CA) by weight. This design focuses on the isolation of the effect of GO as an additive to concrete. The different GO contents of 0%, 0.05%, 0.10%, 0.25%, 0.50%, 0.75%, 1%, and 1.5% as concrete additives are denoted as GO5a, GO10a, GO25a, GO50a, GO75a, GO100a, and GO150a.

## 4. Fabrication Process and Testing

**Figure 3:**
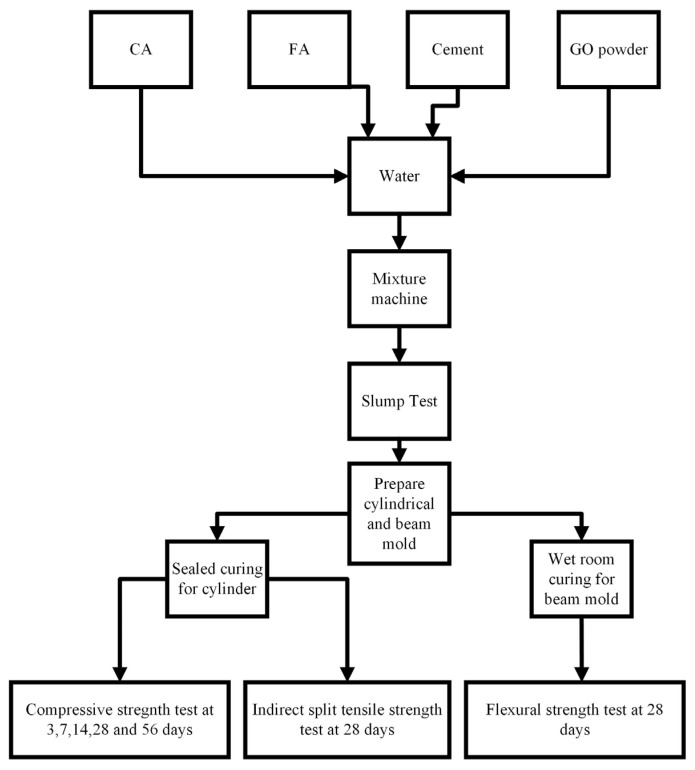
Brief fabrication process of concrete samples and compressive strength testing.

**Figure 4:**
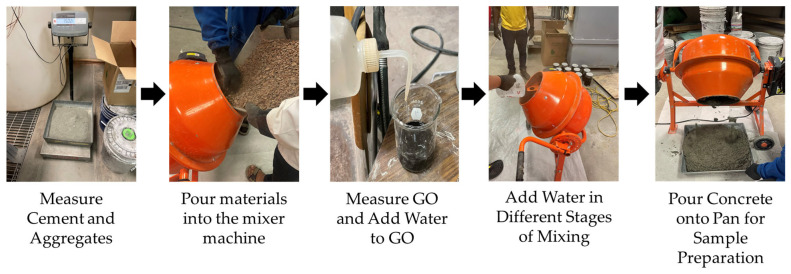
The detailed fabrication process of concrete mix with GO.

Figure 3 and Figure 4 overview the concrete sample fabrication process by following the ACI mix design procedure [41] and subsequent testing for compressive, tensile, and flexural strengths according to different ASTM standards. The CA and FA were then oven-dried for at least 24 h before being incorporated into the concrete samples according to ASTM C192 [53]. The process started by measuring the CA, FA, and cement based on the design mix quantities specified in Table 5 for GO replacement and Table 6 for GO additive. GO was added before pouring into the concrete mix by creating an aqueous solution and mechanically stirred by using an overhead stirrer mixer to maintain its suspension (1000 rpm for 3–4 min) [18]. Figure 3 illustrates the fabrication process of the concrete with GO.

**Figure 5:**
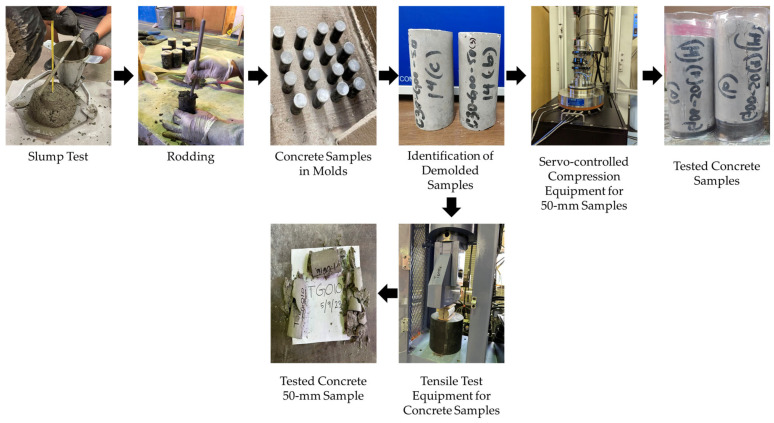
Illustration of slump test, compaction, preparation and demolding of concrete samples, and compressive and tensile testing of concrete samples.

Slump tests were performed Figure 5 on each batch to determine the consistency of freshly mixed concrete before setting, followed by ASTM C143 [54]. Following a slump test, 25 cylindrical concrete samples (50 mm diameter by 100 mm length) were prepared following ASTM C192 [53], as shown in Figure 4. On the designated days for compressive testing (i.e., 3, 7, 14, 28, and 56 days) or tensile strength testing (i.e., 28 days), the concrete samples were carefully removed from the molds using air pressure to minimize any potential damage.

After demolding, all concrete samples intended for the compressive strength test undergo a thorough examination to ensure smoothness and levelness on the top and bottom surfaces. Additionally, each sample’s diameter, length, and mass were carefully measured before conducting the compressive strength test. The density of each concrete mix was calculated by dividing the mass by volume.

The uniaxial compression tests were then conducted using servo-controlled compression system (RTRX-140BX9, GCTS Testing Systems, Tempe, AZ, USA) on the 50-mm diameter concrete samples, following the ASTM C39 [47], as depicted in Figure 5. During the compression testing, axial linear variable differential transformers (LVDTs) and a radial LVDT were incorporated into the GCTS system to measure the axial and radial strains. The stress-strain responses of the 50-mm samples obtained from the compression tests under static loading conditions at 0.1% per min in axial strain were utilized in subsequent analyses to determine both the modulus of elasticity and Poisson’s ratio. For modulus of elasticity testing, the linear portion of the stress-strain curve was determined using tangent moduli.

Figure 5 also illustrates the conduction of the indirect split tensile strength test, following the ASTM C496 [48], using the same 50-mm diameter concrete samples for the three GO percentages of 0.05%, 0.1%, and 0.25%, both as cement replacement and concrete additive by weight of cement, using automatic concrete compression machine (AC-250MRF, Gilson Company, Inc., Lewis Center, OH, USA). This test utilized leather shims on the specimen’s top and bottom. The preload was 2.24 N, and the ramp rate was 1.03 MPa/min until 5% of the sample was broken.

**Figure 6:**
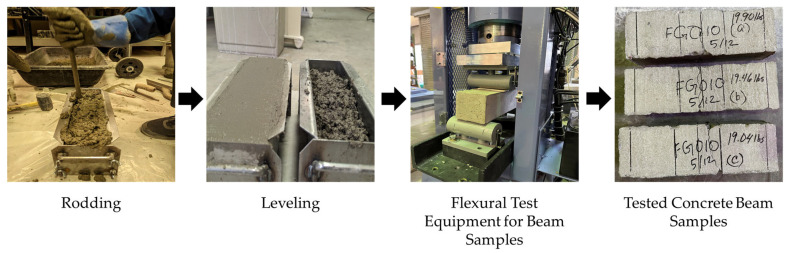
Illustration of preparation of concrete beam samples for flexural strength test of concrete samples.

The flexural strength testing was done for the samples as an indirect split tensile test. For this test, three rectangular concrete beams measuring 100 mm height by 100 mm width by 355 mm length were prepared and tested for flexural strength using the same automatic Gilson AC-250MRF concrete compression machine as for the split tensile strength test, as shown in Figure 6. The test strictly adheres to ASTM C78 [49] ensuring accurate and consistent results. The beams tested under 4-point loading circumstances had their ends strategically supported during the flexure test. The load application started after all prerequisites were met and the configuration was confirmed. The load increased gradually at a constant rate of 1.03 MPa/min until the sample experienced a 15% deformation, resulting in its fracture. In addition, a preload of 2.24 N was used to set the standard for the following loading stages. The average distance on the beam’s tension surface between the fracture line and the closest support was measured after the test.

To ensure statistical accuracy, all mechanical tests—including compressive, flexural, tensile strength, modulus of elasticity, Poisson’s ratio, and density—were performed in triplicate. The only exception was the slump test, which was commonly conducted once per mix.

Heat of hydration tests were conducted on cement paste to observe the hydration rate between cement and water, as well as the influence of GO in the mix. These characteristics of cement-based wet mixtures considering GO as a cement replacement and concrete additive, ranging from 0% to 1.5% by weight of the cement with a water-cement ratio of 0.6, was determined using an isothermal calorimeter (I-Cal 8000 HPC model manufactured by Calmetrix, Arlington, MA, USA) in strict accordance with the guidelines stipulated by ASTM C1702 [55].

To characterize the microstructure, the cement paste samples were initially processed by grinding them into a fine powder. Subsequently, they were sieved to a particle size of 0.075 mm, in preparation for X-ray diffraction (XRD) analysis. XRD patterns were then generated using an X-ray diffractometer (SmartLab, Rigaku Corporation, Tokyo, Japan) with monochromate Cu-Kβ radiation (wavelength of λ = 1.54 Å). The scanning range for 2θ was set from 0 to 60 degrees, with a step size of 0.01 degrees.

Scanning electron microscope (SEM) images and energy dispersive spectroscopy (EDS) data were acquired to visualize the microstructure of paste surfaces and examine the morphology of hydration crystals. These analyses were conducted using a field emission electron microscope (FESEM) (FEI Company, Hillsboro, OR, USA) with an energy-dispersive X-ray spectrometer (Oxford Instruments, Abingdon, Oxfordshire, UK). Notably, the samples intended for EDS analysis were coated with a layer of gold.

## 5. Results and Discussion

### 5.1. Heat of Hydration

**Figure 7:**
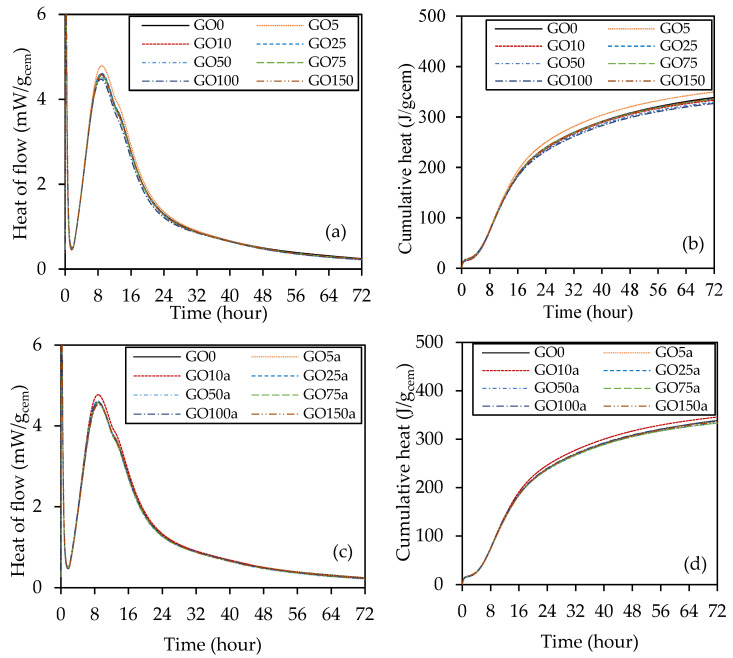
The heat of hydration results: (**a**) heat flow and (**b**) cumulative heat for GO as a replacement, and (**c**) heat flow and (**d**) cumulative heat for GO as an additive.

A calorimetric investigation was carried out on paste samples to examine the effect of coal-derived GO at different percentages in both cases, replacement of cement and additive to concrete, on the cement hydration process within 72 h, as depicted in Figure 7. Figure 7a,b shows the total heat flow and cumulative heat, respectively, for GO as a replacement for cement. It was discovered that with 0.05% GO as a replacement for cement, the hydration rate rose based on the peak increment. In contrast, the other percentages show a minimal increment in the peak but follow the plain cement curve. On the other hand, in Figure 7c,d of the total heat flow and cumulative heat, GO10a shows a higher heat of hydration (increased peak) than the other percentages of GO as an additive. The other percentages display a nearly identical curve when using regular cement paste.

The graphs for all cement pastes, as shown in Figure 7, matched the five stages of a typical Portland cement paste [56]: the initial reaction, induction period, acceleration period, deceleration period, and decline period. It is evident that the addition or removal of peaks was not the result of the incorporation of GO; instead, the peaks’ intensity was altered. In the first 24 h, the heat of hydration rate curve usually shows two peaks. The hydration of C_3_S causes the first peak to evolve, whereas the hydration of the cement’s C_3_A phase is responsible for the second peak. By raising the rate of heat of hydration at the C_3_S and C_3_A hydration phases, GO speeds up the cement hydration process. Similar investigations have found that GO accelerates cement hydration [57]. This is because oxygen-containing functional groups (33.68% oxygen content, Table 1) play a role in cement hydration [58]. As a result of their assertive adsorption behavior [59] through their functional groups, GO can dissolve during the setting phase [60]. During the cement hydration deceleration phase, ions diffuse due to functional groups such as COOH on the GO surface. In addition, during the cement hydration process’s accelerating stage, GO’s functional groups may quickly interact with many ions (Na^+^, K^+^, OH^−^, Ca^2+^) [61]. Due to the nucleation impact, the rate of cement hydration was accelerated by incorporating GO into the cement paste. The nucleation effect caused the GO-cement pastes containing GO to acquire considerably greater hydration heat after eight h of hydration than the plain cement paste. In both cases, the heat flow peaks are higher and moved to the left when comparing the GO composites to the control mix. Additionally, there was a notable intensification in the C_3_A hydration range at around 12 h, especially for GO5 and GO10a. This peak is linked to the revived creation of ettringite [56]. Moreover, the addition of up to 1.5% GO in both cases did not delay or suppress the early exothermic peaks. This suggests that despite the acidic nature of GO, the dosage used in this study did not interfere with hydration kinetics.

Table 7 presents four parameters: age and normalized heat flow at the acceleration phase’s peak and the induction period’s endpoint. It is evident that the samples of GO5 and GO10a increased the heat flow after the induction time by roughly 0.95% and 1.21%, respectively, but did not extend the induction period that much. The prominent hydration peak was achieved by GO10a approximately 0.5% sooner and was raised by 4.23% compared to sample GO0. This indicates that adding GO speeds up cement hydration’s induction and acceleration phases. However, when the hydration time got closer to 48 h, the accumulative hydration heat caused by GO seemed constant till 72 h with the cement paste.

### 5.2. Microstructural Characterization of GO-Concrete Mix

#### 5.2.1. XRD Spectrum Analysis

**Figure 8:**
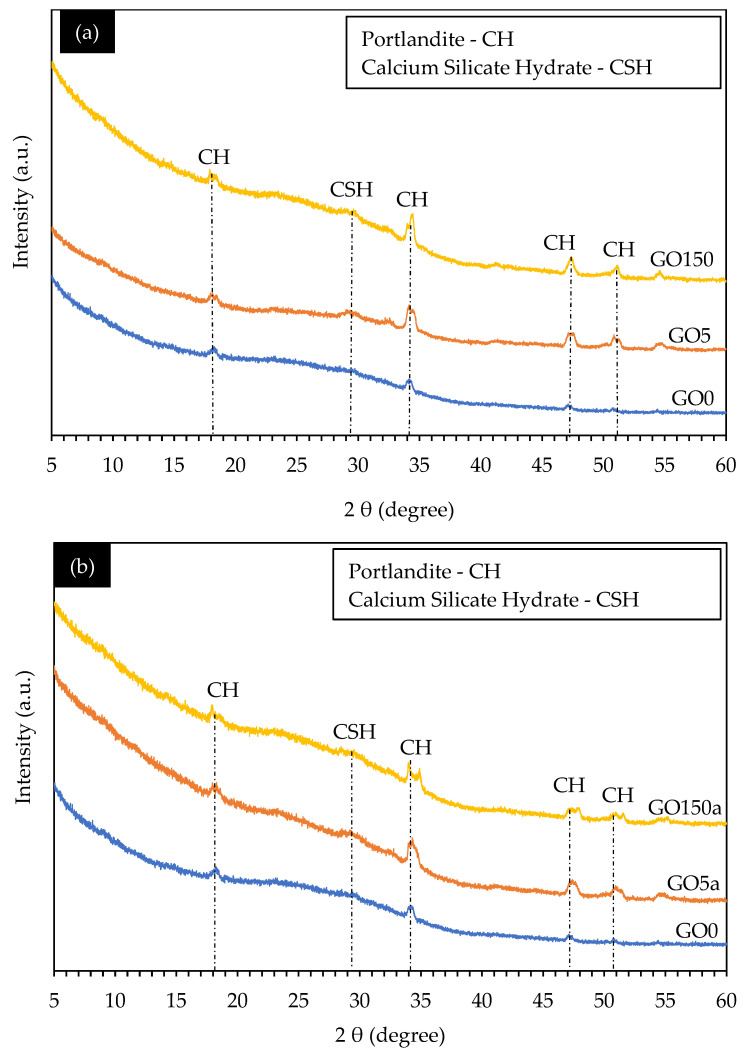
XRD analysis of concrete samples considering (**a**) GO as a cement replacement and (**b**) GO as a concrete additive.

In XRD analysis, using cement paste rather than concrete results in more evident diffraction patterns, improved sensitivity to variations in mixed proportions, and improved curing conditions. This is because, in comparison to concrete, cement paste is more homogenous and displays a higher degree of crystallinity, offering vital information for improving concrete mixtures and comprehending material qualities [62]. Figure 8a,b presents the XRD spectra of selected GO0 and different GO-cement pastes following 56 days of hydration and considering GO as replacement and additive, respectively.

In this work, we aimed to achieve the best possible outcomes by carefully selecting the percentages of GO used. We conducted this investigation in two distinct scenarios: first, where GO was utilized as a substitute for cement, and second, where it was employed as an additive alongside cement. Initially, our starting point was the control mix, which served as the reference point for our experiments. In the subsequent phases of our study, we systematically explored low and high percentages of GO to identify the influence in hydrated products in cement paste. For both cases, we are using 0.05% and 1.5% of GO to understand how the introduction of less or more significant amounts of GO affected the outcome.

In the context of GO-concrete, several sharp peaks suggest an increased content of crystalline phases upon the introduction of GO. This trend aligns with the findings reported by Wang et al. (2017) [63] in studies involving the incorporation of GO into the cement matrix. These sharp peaks were then analyzed to identify specific chemical signals associated with the hydration process, leading to the identification of critical phases such as portlandite (Ca (OH)_2_), and calcium silicate hydrate (CSH) [64].

The heat of hydration results indicated that the hydration phase results were further confirmed and made more evident by the XRD. In both Figure 8a,b, prominent peaks were observed in most of the samples at an angle of 34.3 degrees (2θ), which can be attributed to the presence of portlandite (CH), contributing to rapid early strength development in concrete [65]. Furthermore, two distinct peaks were discerned at 47.15 and 50.80 degrees (2θ), indicative of CH formation.

While the quantity of hydration products in cement paste did not exhibit significant changes with varying GO concentrations, there were fluctuations in their amounts. Specifically, the CSH [66] phase was detected at an angle of 29 degrees (2θ). The intensities of the CSH phase increased with GO, ranging from 0.05% to 1.5% by weight of cement in both replacement and additive cases. This indicates that incorporating GO into cement paste leads to distinct changes in the chemical composition of the hydrated cement matrix. These changes suggest that GO accelerates the hydration process, contributes to the formation of a rich crystalline structure [66], and results in a denser cement matrix. Ultimately, these effects contribute to developing a higher-strength cement matrix, increasing the strength of coal-derived GO concrete.

#### 5.2.2. SEM and EDS Analysis

**Figure 9:**
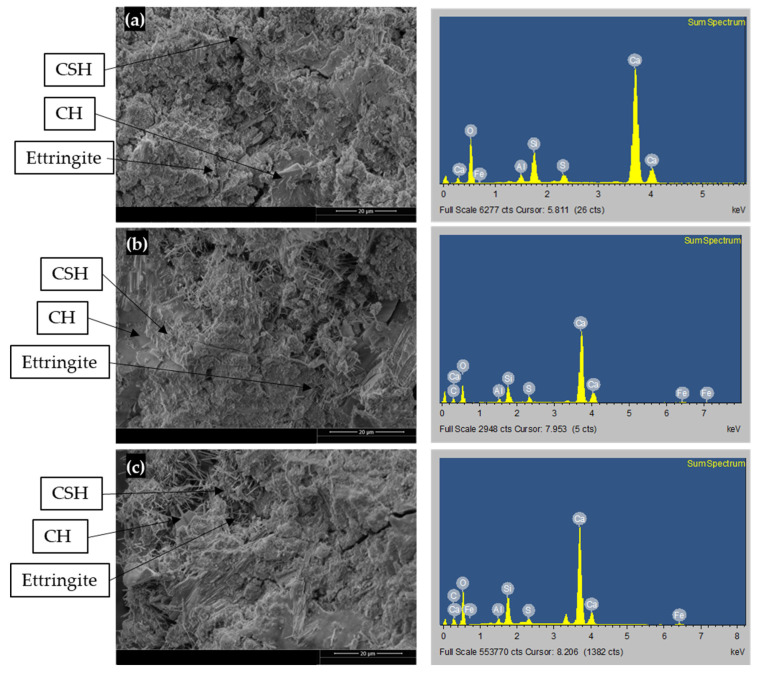
SEM (left) and EDS (right) images: (**a**) C30-GO0-50, (**b**) C30-GO5-50, and (**c**) C30-GO10a-50.

C30-GO5-50 and C30-GO10a-50 were chosen after considering the heat of hydration results and XRD analysis. These tests indicate that the ideal range for the GO percentage lies between 0.05% and 0.10% by weight of cement for both cases. Additional microstructural and mechanical analysis will be conducted to validate this optimal range. Figure 9 presents the SEM micrographs and EDS element analysis of three 56-day cement paste samples, the same sample, which was used for XRD (C30-GO0-50, C30-GO5-50, and C30-GO10a-50) at a fixed magnification level of 20 μm. The microstructure analysis of Figure 9a–c indicates that compared to plain cement paste, the GO-cement paste appeared to have more hydration products and fewer pores. Consequently, the GO helped cement pastes develop more hydration products, which is evident from the heat of hydration and XRD analysis.

Figure 9a (left) displays the microstructure of the control cement paste specimen. From the SEM image, it is possible to observe a loose and non-uniform network structure formation due to the progression of CSH, CH, and needle-like features morphologically consistent with Ettringite crystals [67], along with wide pores and cracks. The SEM pictures of the GO-cement specimens are displayed in Figure 9b (left) and Figure 9c (left), which demonstrates how the addition of GO causes the formation of new crystals of hydrated products to be closely intertwined and have fewer pores and cracks. Compared to the control cement paste, the GO-cement paste surface shows the formation of a more homogeneous, compact, and dense microstructure. This suggests that adding GO to concrete in both cases holds promise as a reinforcing agent [22].

EDS analysis was performed and shown in the right column of Figure 9a–c to investigate the percentage elements of hydrated phases in SEM images. The element analysis from the EDS data, as presented in Table 8, provides valuable insights into the composition of different concrete samples—C30-GO0-50, C30-GO5-50, and C30-GO10a-50. According to the elemental analysis, there was a significant drop in the percentage of components carbon (C), silicon (Si), and calcium (Ca), and an increase in the percentage of oxygen (O) compared to the control sample. In C30-GO0-50, Ca constitutes the majority of the composition at 50.37%, whereas in C30-GO5-50 and C30-GO10a-50, the Ca content decreases to 24.17% and 34.36%, respectively. This suggests that the introduction of GO may impact the distribution of Ca within the concrete matrix. The carbon content, representing the organic component, increases with GO. C30-GO0-50 shows no carbon content, while C30-GO5-50 and C30-GO10a-50 exhibit 12.09% and 9.07% carbon, respectively. The presence of carbon is also evident in Figure 9b (right) column and Figure 9c (right) column. The Si content, reflective of the presence of silicate phases, shows a slight decrease from 8.23% in C30-GO0-50 to 6.02% in C30-GO5-50. The sulfur content shows that C30-GO0-50 has the highest sulfur content at 2.34%, while C30-GO5-50 and C30-GO10a-50 exhibit lower sulfur content. The aluminum and iron content remain relatively consistent across the samples, indicating that GO does not significantly alter the levels of these elements. The oxygen content is noteworthy, increasing from 35.17% in C30-GO0-50 to 53.37% in C30-GO5-50. This increase could be associated with the oxygen-containing functional groups present in GO.

The Ca/Si ratio is a critical parameter in concrete analysis. In the control cement paste, the percentage elemental ratio of Ca/Si was high, and the addition of GO in both cases reduced the Ca/Si ratio, a decrease from 6.12 in C30-GO0-50 to 4.01 in C30-GO5-50. This suggests that during the hydration process in the presence of coal-derived GO, the synthesis of CSH and other hydrated phases interwoven and improved the strength qualities [68]. A similar result was found by Devi and Khan (2020) [22] that the Ca/Si ratio of 0.2 containing 0.08% GO was lower than other mixes with varying GO contents of 0%, 0.02%, 0.04%, and 0.06%, with Ca/Si ratio higher than 2.0. They concluded that this lower Ca/Si ratio promoted the hydration of unreacted cement particles in the later stage of the process. This unique hydration pattern positively influenced the mechanical strength and durability of the resulting material.

### 5.3. Concrete Consistency

**Figure 10:**
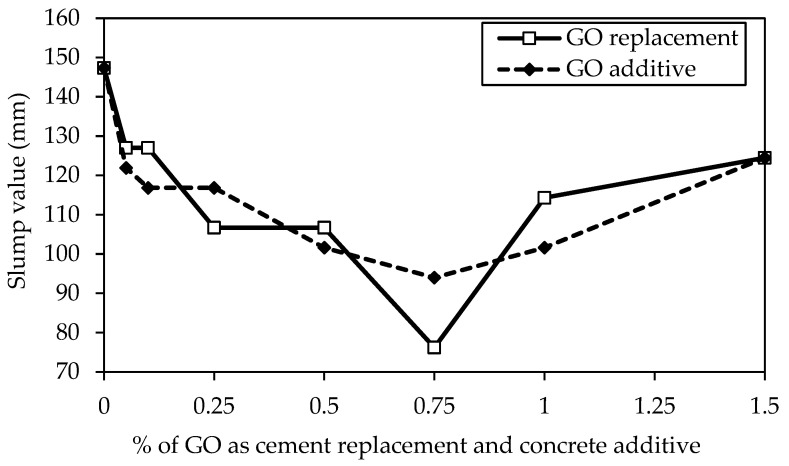
Comparison of slump values of different GO percentages as a replacement and additive by weight of cement.

Figure 10 compares the slump values of the coal-derived GO-concrete, considering GO as a partial cement replacement and concrete additive. It is observed that increasing the percentage of GO content decreases the slump values of the GO-concrete to 0.75% of GO. Compared to the control mix containing 0% GO, it was found that the GO75 and GO75a mix with 0.75% had a substantially smaller lump, trailed by 0.5%, 0.25%, 0.1%, and 0.05% of GO in both cases. Therefore, adding GO as a replacement to cement and additive to concrete mixes decreases their workability. Similar findings on the decrease in fluidity and rise in viscosity with an increase in GO content have been documented [57,69]. Water from freshly mixed concrete is absorbed by the large specific surface area of GO (predicted value of 700–800 m^2^/g) to wet GO [70].

On the other hand, water molecules are absorbed by the hydrophilic oxygenated functions linked to high percentages of GO over 0.75% in both cases and remain stuck due to flocculation and agglomeration formation brought on by the electrostatic contact between GO and cement particles [16,71]. As a result, the mixes’ slump values increase linearly as the GO content rises.

### 5.4. Density

**Figure 11:**
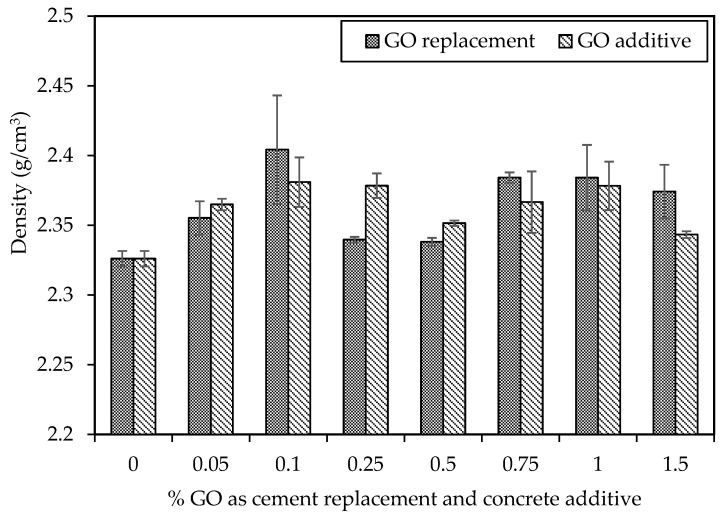
Density of different percentages of GO as a replacement and additive by weight of cement.

Figure 11 illustrates the average density of each concrete mixture after a 28-day curing period, considering GO as a replacement for cement and an additive to concrete. The densities across various mix designs exhibit similarities, ranging between 2.33 and 2.40 g/cm^3^. The control mix shows a 2.33 g/cm^3^ density, while C30-GO10-50 exhibits the highest density at 2.40 g/cm^3^, representing a 3% increase compared to the control mix. The remaining mixes also demonstrate densities surpassing the control mix of 2.33 g/cm^3^. Research suggests that GO can enhance the hydration process and reduce the porosity of cement paste, thereby increasing concrete density. In a specific study, adding 0.05% GO resulted in a 4.76% increase in concrete density compared to plain concrete [72]. Another study observed a 5.6% density increase when 0.1% GO was added to concrete [73]. These findings propose that GO may act as a nucleation site for hydration products, such as CSH and calcium aluminate hydrate (CAH), filling pores and increasing the overall concrete density.

### 5.5. Uniaxial Compressive Strength (UCS)

**Figure 12:**
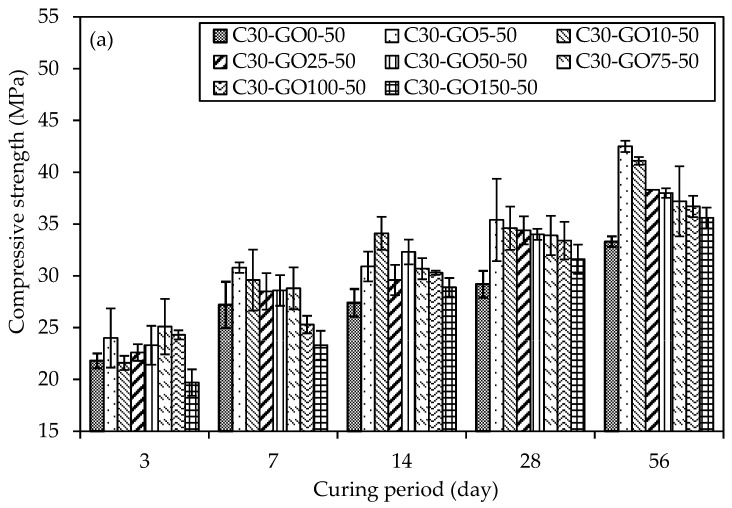

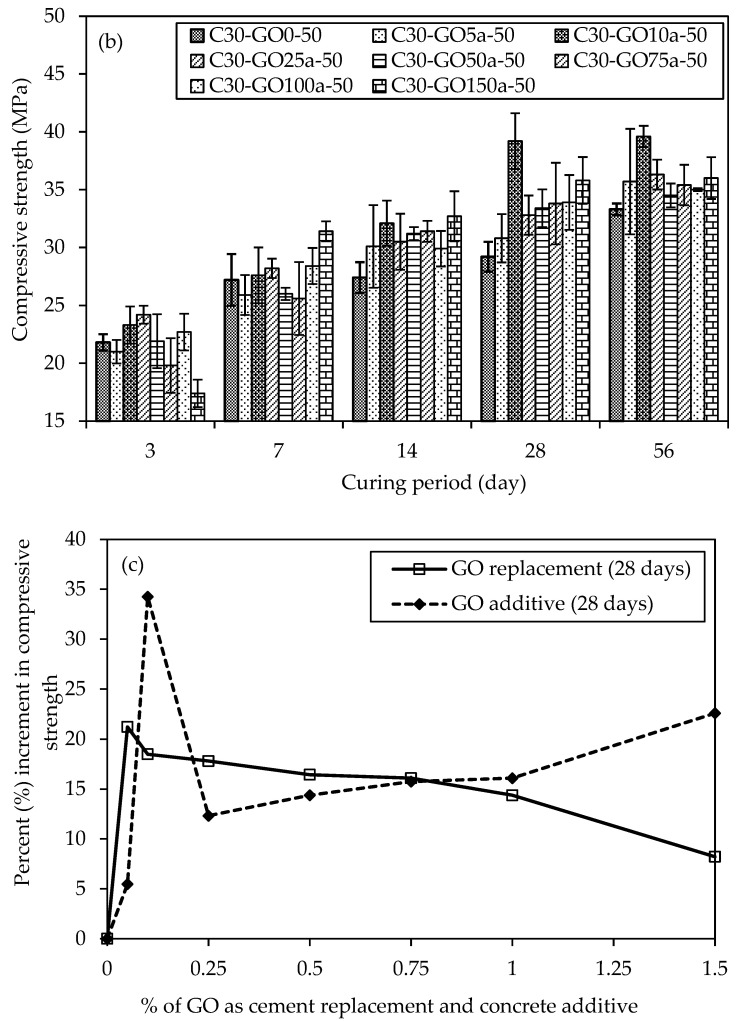

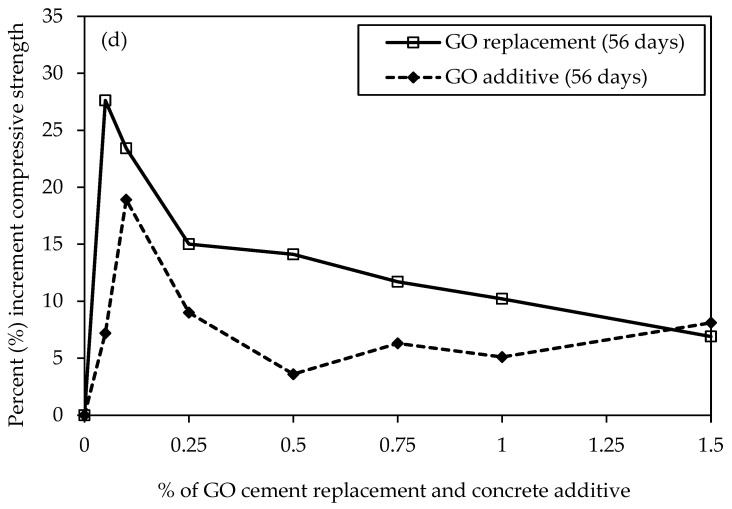
Comparison of uniaxial compressive strength and percentage of GO of concrete samples in different curing periods (**a**) GO as a replacement and (**b**) GO as an additive, and percent (%) increment of compressive strength for both cases of GO, (**c**) 28-day curing period and (**d**) 56-day curing period.

Figure 12a,c represent GO as a replacement, show the UCS results with percent increment for various concrete mixtures with varying percentages of GO subjected to different curing periods. On three days, C30-GO5-50 displays an early strength advantage with a 10.1% increment compared to the control mix, C30-GO0-50. As the curing duration extends to seven days, the positive trend continues, showcasing a 13.2% increase in strength for C30-GO5-50. Notably, in 14 days, the compressive strength increment reached 12.8%, indicating continued enhancement attributed to 0.05% GO. However, for C30-GO10-50, the 14-day mark marks a significant increase, with a 24.5% strength increment, surpassing the 0.05% GO mix. As the curing progresses to 28 days, all mixes experience strength gains, with C30-GO5-50 leading to a 21.2% increment over the control. Over 56 days, C30-GO5-50 achieves the highest strength of 42.5 MPa, showcasing a remarkable 27.6% increase. This result highlights the long-term efficacy of 0.05% GO in increasing compressive strength, underscoring the importance of taking concentration and curing time into account. Conversely, higher GO concentrations in C30-GO10-50, C30-GO25-50, C30-GO50-50, C30-GO75-50, and C30-GO100-50 exhibit varying degrees of improvement, suggesting a nuanced interplay between GO content and compressive strength. C30-GO10-50 exhibits a remarkable 18.5% strength increment at 28 days, reaching 34.6 MPa, and maintains the strength gain at 56 days, achieving a final strength of 41.1 MPa, marking a 23.4% increase. C30-GO25-50 exhibits the effect of 0.25% GO, with a 17.8% strength increment at 28 days and a final strength of 38.3 MPa at 56 days. Meanwhile, C30-GO50-50 reveals a 16.4% strength increment at 28 days and maintains an absolute strength of 38.0 MPa at 56 days, affirming the continued positive influence of 0.50% GO. C30-GO75-50 shows an 11.7% strength increment at 56 days, while C30-GO100-50 exhibits a 10.2% increment, reaching a final strength of 36.7 MPa. However, C30-GO150-50 shows a decrease in strength at 56 days, emphasizing the importance of optimal GO concentration for maximal strength benefits.

Moreover, the UCS results with percent increment are shown in Figure 12b,d GO as an additive to concrete. At three days, C30-GO5a-50 exhibits a compressive strength of 21.0 MPa, while C30-GO10a-50 and C30-GO25a-50 demonstrate higher strengths at 23.3 MPa and 24.2 MPa, respectively. The control mix, C30-GO0-50, demonstrates competitive strength at 21.80 MPa, indicating that GO inclusion may impact the immediate strength gain at the early stage, which is also evident from the heat of the hydration curve. As curing progresses, C30-GO10a-50 experiences a substantial increase in strength, reaching 39.2 MPa in 28 days, marking a 34.2% increment compared to the control. This upward trend continues at 56 days, where C30-GO10a-50 maintains the highest strength among all mixtures at 39.6 MPa. Conversely, C30-GO5a-50 exhibits a consistent increase in strength, reaching 35.7 MPa at 56 days, showcasing a 7.2% increment from 28 days. As GO concentrations increase to 0.50% and beyond, the compressive strength of C30-GO50a-50, C30-GO75a-50, C30-GO100a-50, and C30-GO150a-50 demonstrates mixed outcomes. C30-GO50a-50 displays 34.5 MPa at 56 days, marking a 14.4% increase from 28 days. Conversely, C30-GO75a-50 and C30-GO100a-50 exhibit fluctuations, with strengths at 56 days slightly decreasing by 9.5% and 11%, respectively, compared to 28 days. C30-GO150a-50 demonstrates a 106.9% strength increase at 56 days, reaching 36.0 MPa from 17.4 MPa at three days, showcasing a different trend among the studied mixtures. This comparative analysis underscores that C30-GO10a-50 exhibits the highest strength.

The varying optimal GO percentages for early strength gain, peak strength at 28-day, and 56-day strength relate to GO’s role at different hydration stages in the concrete mix. In the initial stages, GO may not contribute significantly to early strength gain compared to the control mix, as indicated by the heat of hydration curves up to 72 h Figure 7a,b. The consistency in optimal percentages between 28 and 56 days Figure 12c,d suggests that GO’s impact on strength becomes more pronounced in later hydration stages, potentially through improved bonding with cement hydration products and enhancing the overall long-term strength and durability of the concrete structure. Drawing insights from the UCS above results, it becomes apparent that the optimal concentration of GO falls within the range of 0.05% to 0.10% in both replacement and additive scenarios. This conclusion aligns with corroborating evidence obtained from heat of hydration and microstructural characterization tests. The heat of hydration analysis reveals a notable increase in the peak when GO concentration is within the 0.05% to 0.10% range, indicating enhanced hydration acceleration and the formation of more hydrated products. Furthermore, microscopic examinations, such as XRD and SEM, substantiate these findings by demonstrating an increased presence of hydrated products within the same concentration range.

To understand whether the observed strength gains were meaningful, we performed two-sample t-tests comparing the control mix with GO-modified concretes at 28 and 56 days, under both replacement and additive conditions. The statistical significance was assessed at a 95% confidence level. As shown in Table 9, the mixes with 0.10% GO demonstrated statistically significant improvements in most cases, particularly at 56 days. This indicates that even at lower dosages, GO contributes meaningfully to strength enhancement.

### 5.6. Flexural and Split Tensile Strength

**Figure 13:**
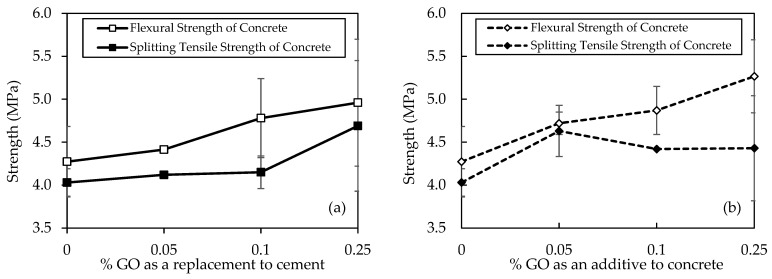
Flexural and tensile strengths of concrete samples for (**a**) GO as a replacement to cement and (**b**) GO as an additive to concrete.

Figure 13a shows the flexural and split tensile strength results for various concrete mixtures, denoted by different concentrations of GO as a replacement and subjected to 28 days of curing, providing a detailed insight into the mechanical properties of the specimens. In terms of flexural strength, the progression is notable, with C30-GO0-50 exhibiting the lowest strength at 4.27 MPa, followed by incremental improvements in C30-GO5-50 (4.41 MPa), C30-GO10-50 (4.8 MPa), and C30-GO25-50 (5.0 MPa). This sequential increase suggests a positive correlation between GO concentration and flexural strength. C30-GO25-50 stands out as the mix with the highest flexural strength, demonstrating a 16.1% increase compared to the control mix, C30-GO0-50. Similarly, a similar trend is observed in terms of split tensile strength. C30-GO0-50 displays the lowest split tensile strength at 4.03 MPa, followed by gradual enhancements in C30-GO5-50 (4.12 MPa), C30-GO10-50 (4.2 MPa), and reaching the highest strength in C30-GO25-50 at 4.7 MPa. Compared to the control, the percentage increments in split tensile strength are consistent with the flexural strength trends. C30-GO25-50, with a 16.4% increase, again emerges as the mix with the highest split tensile strength.

However, Figure 13b shows GO’s split tensile and flexural strengths added to concrete at different percentages. In terms of flexural strength, a clear ascending trend is observed. The control mix, C30-GO0-50, demonstrates a flexural strength of 4.27 MPa, while incremental improvements are evident in C30-GO5a-50 (4.72 MPa), C30-GO10a-50 (4.9 MPa), and reaching the highest strength in C30-GO25a-50 at 5.3 MPa. This progression underscores a positive correlation between GO concentration and flexural strength, with C30-GO25a-50 exhibiting a significant 23.2% increase compared to the control. Similarly, the split tensile strength results align with this trend. C30-GO0-50 displays a split tensile strength of 4.03 MPa, followed by enhancements in C30-GO5a-50 (4.63 MPa), C30-GO10a-50 (4.4 MPa), and parity in C30-GO25a-50 (4.4 MPa). The improvement in split tensile strength is most pronounced in C30-GO5a-50, with a 14.9% increase compared to the control.

The observed improvements in flexural and split tensile strength can be attributed to the reinforcing effects of GO that facilitate the formation of hydrated CSH, which enhances the bonding between the cementitious matrix and aggregates [74]. The 28-day curing period allows for the formation of a well-developed structure, and the introduction of GO facilitates the creation of a more interconnected network within the concrete [75]. The increasing concentrations of GO result in a progressive strengthening effect, as evidenced by the higher mechanical properties observed in 0.05% and 0.10%, and eventually peaking in 0.25% of GO in both cases.

### 5.7. Modulus of Elasticity (E) and Poisson’s Ratio

**Figure 14:**
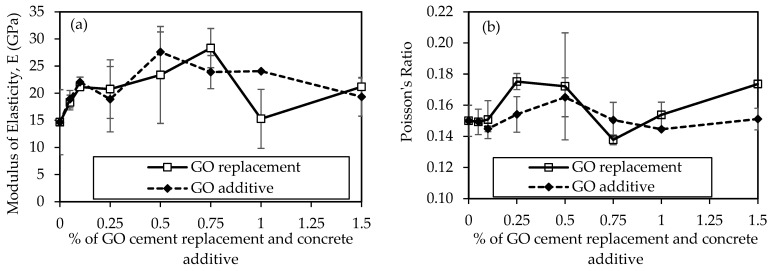
Comparison of some mechanical properties of concrete samples using GO at different percentages of cement: (**a**) Moduli of elasticity, and (**b**) Poisson’s ratio.

Figure 14a compares the modulus of elasticity (E) results for concrete mixtures, considering GO as a replacement and an additive at various percentages from 0% to 1.5%, unveiling insightful trends in the material’s elasticity over a 28-day curing period. In the context of GO as a replacement, the control mix (0% GO) exhibits an E of 14.70 GPa. The introduction of 0.05% GO results increase to 18.29 GPa, marking a 24.4% improvement. The upward increment continues with 0.1% GO, reaching 21.13 GPa, indicating a further 43.7% increase. However, as the GO concentration rises to 0.25%, a slight decrease is observed, settling at 20.75 GPa. The trend shifts again with 0.5% GO, showcasing the highest E at 0.75% of GO as 28.31 GPa, a significant 92.6% increase compared to the control mix. In contrast, when GO is introduced as an additive, adding 0.05% GO results in an E of 19.30 GPa, surpassing the equivalent replacement scenario and indicating a 31.3% increase. This positive trend persists with 0.1% GO, reaching 22.0 GPa, marking a 49.9% increase. The E then experiences a slight decrease at 0.25% GO, settling at 18.9 GPa, yet still surpassing the equivalent replacement concentration. However, the most notable increase is observed with 0.5% GO, reaching 27.6 GPa, showcasing a remarkable 87.8% improvement compared to the control mix.

The Poisson’s ratio results for concrete mixtures are illustrated in Figure 14b. Poisson’s ratio is a dimensionless parameter indicating transverse contraction to longitudinal extension ratio. In both scenarios, where GO is used as a replacement to cement and additive to concrete, it shows variations across different concentrations. This reveals that Poisson’s ratio remains relatively constant (around 0.15) for lower concentrations (0%, 0.05%, 0.1%) but experiences variations at higher concentrations (0.25%, 0.5%, 0.75%, 1%, 1.5%). The values range from 0.14 to 0.19. This suggests that replacing a portion of the cement with GO does not significantly alter the lateral contraction behavior of the concrete, maintaining a uniform response across the tested concentrations. Typically, the Poisson’s ratio in regular concrete falls between 0.15–0.25 [76], indicating our results align with this range. However, 0.5% GO in both cases exhibits the highest Poisson’s ratio at 0.19, indicating some tendency for lateral contraction compared to other concentrations.

The observed trends in E and Poisson’s ratio can be attributed to the reinforcing effects of GO in the concrete matrix. The optimal concentration varies between replacement and additive scenarios, with 0.5% GO showing the highest E in the additive case and the highest Poisson’s ratio in both cases. Subsequent concentrations (0.75%, 1%, and 1.5%) exhibit varying degrees of increase and decrease, indicating complex interactions between GO content and E and Poisson’s ratio. Some studies suggest that GO can modify the microstructure and hydration of concrete, affecting the concrete’s modulus of elasticity and Poisson’s ratio. By improving the hydration process and decreasing the porosity of the concrete, adding GO to concrete can raise its modulus of elasticity [77] and Poisson’s ratio [78]. This is because GO may serve as a nucleation site for hydration products that can fill in the pores and raise the mass of the concrete [79]. Furthermore, GO can strengthen its bonds by enhancing the cement particles’ surface area and decreasing their friction [80]. On the contrary, fluctuations at higher concentrations may indicate a saturation point or other complex interactions between GO and the concrete constituents. By interfering with the hydration process and increasing the number of air voids in the concrete, adding excessive amounts of GO to concrete can lower its modulus of elasticity and decrease Poisson’s ratio [72]. This is because GO can obstruct the hydration process by enveloping CSH or CAH crystals in a protective shell that keeps them from developing or coming into balance with water molecules [81].

## 6. Conclusions

In this study, we conducted a detailed experimental analysis to understand how low-cost coal-derived GO enhances concrete qualities. By incorporating this GO in different proportions, we investigated its impact on concrete’s mechanical properties and microstructure. The results highlighted the following key points:

- Heat of hydration: Mixtures with specific coal-derived GO concentrations (GO5 and GO10a) exhibited significantly higher heat of hydration, indicating active interaction within the concrete mix and contributing to enhanced strength.
- Microstructural changes: Analysis of XRD and SEM images revealed that adding coal-derived GO influenced the configuration and dispersion within the concrete matrix. This led to a more uniform and compact structure, enhancing strength and resilience.
- Ca/Si ratio: The Ca/Si ratio in GO5 and GO10a was lower than that in the control mix, indicating further evidence of strengthened properties due to coal-derived GO incorporation.
- Enhanced strength: When coal-derived GO was partially substituted or added to cement, there was a significant increase in flexural, split tensile, and compressive strengths compared to the control mix, especially at the 56-day mark. The strength improvements became more pronounced with lower GO content, reaching a remarkable 27.6% increase in compressive strength when GO replaced cement at 0.05 wt.%.
- Mechanical performance: Measurements of the modulus of elasticity and Poisson’s ratio demonstrated substantial strength gains due to coal-derived GO incorporation, indicating enhanced mechanical performance.
- Optimal concentration: The study identified the optimal coal-derived GO concentration range (0.05 to 0.10 wt.%) for maximum compressive strength. Within this range, the concrete exhibited superior durability and resilience, indicating the effective strengthening potential of GO.

In summary, our comprehensive study demonstrated significant improvements in various strength parameters, supported by multiple lines of evidence such as modulus of elasticity, Poisson’s ratio, heat of hydration, and microstructural analyses. These findings emphasize the effectiveness of coal-derived GO in enhancing the strength of concrete. Concrete durability in terms of chloride ion penetration, permeability, and sulfate resistance is being conducted and will be reported in future publications.

## 7. Patents

This paper is part of the patent document No. WO2024155700A3.

## Figures and Tables

**Table 1:** Study on compressive strength from different sources.

References	Addition of GO by Weight (%)	Water-Cement Ratio	Compressive Strength (MPa)		Percent Increment (%)
			Control Mix	with GO-Concrete	
(Wu et al., 2019) [4]	0.03	0.50	41.18	55.20	34.00
(Du et al., 2019) [20]	0.10	0.40	54.20	84.50	56.00
(Chen et al., 2020) [21]	0.05	0.35	56.00	64.00	14.00
(Devi & Khan, 2020) [22]	0.06	0.45	32.00	40.00	25.00
(Wu et al., 2020) [23]	0.03	0.16	97.00	120.00	24.00
(Zheng et al., 2020) [24]	0.06	0.10	122.00	140.00	15.00
(Chen et al., 2019) [25]	0.08	0.35	56.90	64.10	13.00
(Yuan, et al., 2022) [26]	0.03	0.34	58.20	61.40	5.00

**Table 2:** Properties of coal-derived and commercial GO.

Characterization	Coal-Derived GO	Commercial GO
Lateral size (μm)	0.30–1.00 [30]	0.2–10
Shape	Micron-sized powders	Nano sheet
pH	~3.00	~3.57
Carbon content (wt.%)	61.91	~42.70
Oxygen content (wt.%)	33.68	~51.60
Sulfur content (wt.%)	-	<2.10
Carbon/Oxygen ratio	1.84	0.83

**Table 3:** Chemical composition analysis of Quikrete OPC (Type I/II).

Components	Mass (%)
Silicon Dioxide (SiO_2_)	19.66
Calcium Oxide (CaO)	63.33
Sulfur Trioxide (SO_3_)	3.62
Aluminium Oxide (Al_2_O_3_)	4.36
Iron Oxide (Fe_2_O_3_)	3.41
Magnesium Oxide (MgO)	1.22
Sodium Oxide (Na_2_O)	0.13
Potassium Oxide (K_2_O)	0.81

**Table 4:** Properties of CA and FA.

Property	Standard	CA	FA
Maximum Aggregate Size (mm)	ASTM C33 [42]	25.00	4.75
Minimum Aggregate Size (mm)		2.36	0.15
Bulk Specific Gravity (OD)	ASTM C127 [43]	2.71	2.59
Bulk Specific Gravity (SSD)		2.71	2.57
Apparent Specific Gravity		2.72	-
Dry rodded Unit Weight (kg/m^3^)	ASTM C29 [44]	1590.73	1586.68
Absorption Capacity (%)	ASTM C127 [43]	0.16	0.60
Moisture Content (%)	ASTM C566 [45]	1.00	3.20
Fineness modulus (FM)	ASTM C136 [46]	7.78	2.58

**Table 5:** Mix design of concrete (by weight) with GO as a cement replacement.

Designation	GO Replacement Percentage (%)	Water (kg/m^3^)	Cement (kg/m^3^)	GO (kg/m^3^)	FA (kg/m^3^)	CA (kg/m^3^)
C30-GO0-50	0.00	228.00	380.00	0.00	876.00	788.00
C30-GO5-50	0.05	228.00	379.81	0.19	876.00	788.00
C30-GO10-50	0.10	228.00	379.62	0.38	876.00	788.00
C30-GO25-50	0.25	228.00	379.05	0.95	876.00	788.00
C30-GO50-50	0.50	228.00	378.10	1.90	876.00	788.00
C30-GO75-50	0.75	228.00	377.15	2.85	876.00	788.00
C30-GO100-50	1.00	228.00	376.20	3.80	876.00	788.00
C30-GO150-50	1.50	228.00	374.30	5.70	876.00	788.00

**Table 6:** Mix design of concrete (by weight) with GO as a concrete additive.

Designation	GO Additive Percentage (%)	Water (kg/m^3^)	Cement (kg/m^3^)	GO (kg/m^3^)	FA (kg/m^3^)	CA (kg/m^3^)
C30-GO5a-50	0.05	228.00	380.00	0.19	876.00	788.00
C30-GO10a-50	0.10	228.00	380.00	0.38	876.00	788.00
C30-GO25a-50	0.25	228.00	380.00	0.95	876.00	788.00
C30-GO50a-50	0.50	228.00	380.00	1.90	876.00	788.00
C30-GO75a-50	0.75	228.00	380.00	2.85	876.00	788.00
C30-GO100a-50	1.00	228.00	380.00	3.80	876.00	788.00
C30-GO150a-50	1.50	228.00	380.00	5.70	876.00	788.00

**Table 7:** Heat flow in different phases.

Sample Name	Minimum Age During the Induction Phase (h)	Lowest Heat Flow During the Induction Phase (mW/g)	% Increment of the Lowest Heat Flow	Peak Heat Flow Age (h)	Peak Heat Flow (mW/g)	% Increment of the Peak Heat Flow
GO0	1.65	0.47	-	8.87	4.58	-
GO5	1.67	0.47	0.95%	8.87	4.79	4.54%
GO10a	1.65	0.48	1.21%	8.82	4.77	4.23%

**Table 8:** Element analysis from EDS data.

Elements	C30-GO0-50	C30-GO5-50	C30-GO10a-50
Ca	50.37	24.17	34.36
C	0.00	12.09	9.07
Si	8.23	6.02	5.75
S	2.34	1.99	1.14
Al	1.78	1.64	1.16
Fe	2.11	0.72	1.07
O	35.17	53.37	47.46
Total	100.00	100.00	100.00
Ca/Si Ratio	6.12	4.01	5.98

**Table 9:** Statistical analysis of compressive strength for control and GO-modified concrete at 28 and 56 days.

Comparison Group	Scenario	Age (days)	Mean Strength (MPa)	Standard Deviation (MPa)	*p*-Value	Significance
Control vs. 0.05% GO	Replacement	28	29.2 vs. 35.4	1.29/3.98	0.062	Not significant
Control vs. 0.10% GO	Replacement	28	29.2 vs. 34.6	1.29/2.08	0.019	Significant
Control vs. 0.05% GO	Replacement	56	33.3 vs. 42.5	0.51/0.54	<0.001	Significant
Control vs. 0.10% GO	Replacement	56	33.3 vs. 41.1	0.51/0.37	<0.001	Significant
Control vs. 0.05% GO	Additive	28	29.2 vs. 30.8	1.29/2.09	0.322	Not significant
Control vs. 0.10% GO	Additive	28	29.2 vs. 39.2	1.29/2.41	0.003	Significant
Control vs. 0.05% GO	Additive	56	33.3 vs. 35.7	0.51/4.56	0.416	Not significant
Control vs. 0.10% GO	Additive	56	33.3 vs. 39.6	0.51/0.92	0.001	Significant

## Data Availability

The original contributions presented in this study are included in the article. Further inquiries can be directed to the corresponding author.

## Abbreviations

The following abbreviations are used in this manuscript:

GDGO	Graphite-Derived Graphene Oxide
GO	Graphene Oxide
CSH	Calcium Silicate Hydrate
CH	Calcium Hydroxide/Portlandite
PRB	Powder River Basin
OPC	Ordinary Portland Cement
CA	Coarse Aggregate
FA	Fine Aggregate
FM	Fineness Modulus
OD	Oven-Dry
SSD	Saturated Surface Dry
ACI	American Concrete Institute
LVDTs	Linear Variable Differential Transformers
ASTM	American Society for Testing and Materials
XRD	X-ray Diffraction
SEM	Scanning Electron Microscope
EDS	Energy Dispersive Spectroscopy
C_3_S	Tricalcium Silicate
C_3_A	Tricalcium Aluminate
CAH	Calcium Aluminate Hydrate
E	Modulus Of Elasticity
